# Security Analysis of a Color Image Encryption Algorithm Using a Fractional-Order Chaos

**DOI:** 10.3390/e23020258

**Published:** 2021-02-23

**Authors:** Heping Wen, Chongfu Zhang, Lan Huang, Juxin Ke, Dongqing Xiong

**Affiliations:** 1 School of Electronic and Information, Zhongshan Institute, University of Electronic Science and Technology of China, Zhongshan 528402, China; cfzhang@uestc.edu.cn (C.Z.); greentree_2001@163.com (L.H.); 2 School of Information and Communication Engineering, University of Electronic Science and Technology of China, Chengdu 611731, China; 3 Center of Information and Technology, Dongguan Polytechnic, Dongguan 523808, China; kejx@dgpt.edu.cn; 4 Guangdong Mechanical and Electronical College of Technology, Guangzhou 510515, China; xiongdongqing@gdmec.edu.cn

**Keywords:** chaos, image encryption, cryptanalysis

## Abstract

Fractional-order chaos has complex dynamic behavior characteristics, so its application in secure communication has attracted much attention. Compared with the design of fractional-order chaos-based cipher, there are fewer researches on security analysis. This paper conducts a comprehensive security analysis of a color image encryption algorithm using a fractional-order hyperchaotic system (CIEA-FOHS). Experimental simulation based on excellent numerical statistical results supported that CIEA-FOHS is cryptographically secure. Yet, from the perspective of cryptanalysis, this paper found that CIEA-FOHS can be broken by a chosen-plaintext attack method owing to its some inherent security defects. Firstly, the diffusion part can be eliminated by choosing some special images with all the same pixel values. Secondly, the permutation-only part can be deciphered by some chosen plain images and the corresponding cipher images. Finally, using the equivalent diffusion and permutation keys obtained in the previous two steps, the original plain image can be recovered from a target cipher image. Theoretical analysis and experimental simulations show that the attack method is both effective and efficient. To enhance the security, some suggestions for improvement are given. The reported results would help the designers of chaotic cryptography pay more attention to the gap of complex chaotic system and secure cryptosystem.

## 1. Introduction

Nowadays, with the rapid development of optical fiber broadband access network, 5G and other communication technologies, the security of multimedia data, especially digital images, is of particular interest in communication networks [1]. As everyone knows, encryption is an effective means of achieving security enhancements [2]. However, traditional text encryption algorithms such as AES, DES, and IDEA are not suitable for digital images because they featured with strong correlation between adjacent pixels. To deal with the problem, various methodologies are introduced to design different image ciphers. Among them, chaos-based image encryption is the most popular one, because chaos has characteristics of sensitivity to initial values, dense periodic points, and long-term unpredictability of orbits [3,4,5]. In the past two decades, chaotic image encryption technology has been widely discussed and has become a research hotspot [6]. To improve the security performance of chaotic image encryption technology, various chaotic systems with resistance to dynamic degradation are studied, including quantum chaotic map [7], fractional-order chaos [8], non-degenerated hyperchaos [9], economic chaotic map [10], and cascaded chaotic systems [11], etc. However, chaotic cryptography still lacks authoritative metrics, especially in terms of security. Accordingly, many reported chaotic encryption algorithms have been broken [12,13,14,15]. As shown in Table 1, some previous chaos-based ciphers are vulnerable upon various attack methods, including chosen-ciphertext attack [16], chosen-/known-plaintext attack [12], differential cryptanalysis [17], even cipher-only attack [18]. Therefore, research on security is extremely important and has received much attention [19,20,21,22,23,24,25,26,27,28,29,30,31,32,33].

As described in Ref. [39], fractional-order chaotic systems have higher complexity and more optional key parameters and can be used as a competitive encryption scheme. Correspondingly, image encryption algorithms based on fractional-order chaotic systems have attracted the attention of researchers in recent years [35,40,41,42]. In 2013, Wang et al. [40] introduced a fractional-order chaos into image encryption for the first time, and gave some experiments to verify its performance. Since then, many image encryption schemes based on fractional-order chaotic systems have been proposed [35,41,42]. For example, in 2017, Zhang et al. [41] proposed a color image encryption scheme combing with fractional-order hyperchaotic system and DNA encoding. Yet, cryptanalysts have reported that some fractional-order chaotic image encryption algorithms have some fatal security issues. Exactly, Norouzi et al. [36] pointed out that the image cipher that using an improper fractional-order chaotic system was insecure, which was proposed in [35]. As far as we know, there are still few research studies concerning cryptanalysis on the ciphers based on fractional-order chaotic systems. Moreover, considering that each cryptosystem has its intrinsic characteristics, it is necessary and urgent to perform cryptanalysis on these existing ciphers.

In 2015, a color image encryption algorithm based on a fractional-order hyperchaotic system was proposed [42]. In color image encryption algorithm using a fractional-order hyperchaotic system (CIEA-FOHS), using the pseudo-random sequences generated by the fractional-order hyperchaotic system, RGB-inter permutation, RGB-intra permutation and pixel diffusion are successively performed to get cipher images from plain images. Meanwhile, the relevant pixel correlation, histogram and other experimental analysis are given to verify its security performance. However, from the perspective of cryptanalysis, we found some security defects as follows:

- The existence of an equivalent key. CIEA-FOHS encrypts the image using a pseudo-random sequence generated by fractional-order chaos. However, these sequences are not related to plaintext. Thus, these sequences can be considered as equivalent keys.
- Two-stage permutations can be equivalently simplified to only once. The reason is that the two permutations only change the position of the pixel without changing the value of the pixel.
- The paradigm of the diffusion part is insecure. According to the conclusion of Ref. [43], a class of diffusion encryption using module addition and XOR operations can be cracked with only two special plain images and their corresponding cipher images. Unfortunately, CIEA-FOHS is also the case.

Based on the three points, CIEA-FOHS cannot resist against a chosen-plaintext attack method with the divide-and-conquer strategy. More specifically, under the scenario of chosen-plaintext attack, firstly an equivalent diffusion key is obtained, and then an equivalent permutation key is achieved, and finally the original images can be restored from the encrypted images with the equivalent keys.

## 2. The Encryption Algorithm under Study

In this section, the fractional-order hyperchaotic system used in Reference [42] is presented, and then the specific steps of CIEA-FOHS are introduced.

### 2.1. Fractional-Order Hyperchaotic System

**Figure 1:**
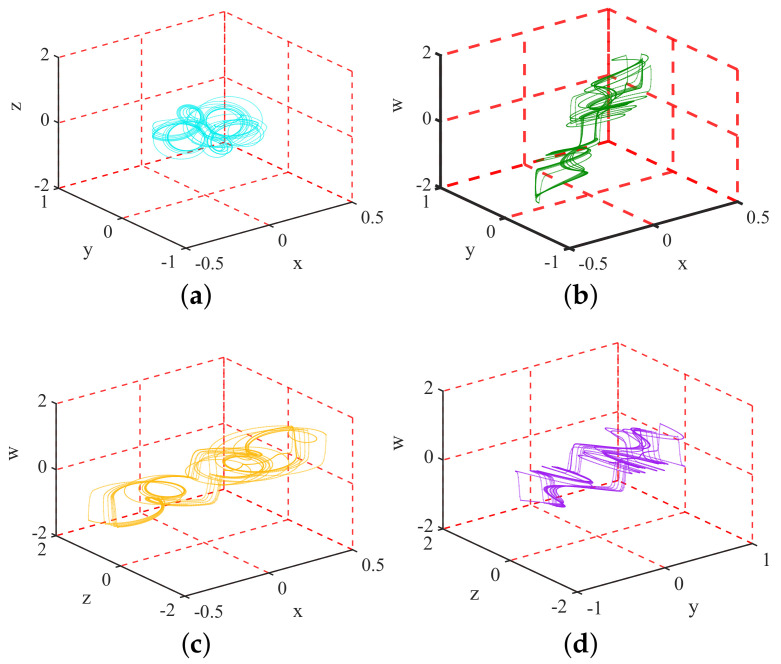
Attractor phase diagrams of the fractional-order hyperchaotic system with different variables: (**a**) (x,y,z); (**b**) (x,y,w); (**c**) (x,z,w); (**d**) (y,z,w).

The fractional-order hyperchaotic system used in CIEA-FOHS is derived from Ref. [39], given as

(1) $$\left\{\begin{array}{c} D_{t}^{\alpha }x\left(t\right)=-z-w\\ D_{t}^{\alpha }y\left(t\right)=2y+z\\ D_{t}^{\alpha }z\left(t\right)=14x-14y\\ D_{t}^{\alpha }w\left(t\right)=100\left(x-g\left(w\right)\right) \end{array}\right.$$

where x,y,z,w are the four state variables, $g\left(w\right)=w-\left(\left|w-0.4\right|-\left|w-0.8\right|-\left|w+0.4\right|-\left|w+0.8\right|\right)$, $D_{t}^{\alpha }$ is the fractional derivative under the definition of Caputo and α is the derivative order. The attractor of the fractional-order hyperchaotic system is shown in Figure 1.

### 2.2. Description of CIEA-FOHS

**Figure 2:**
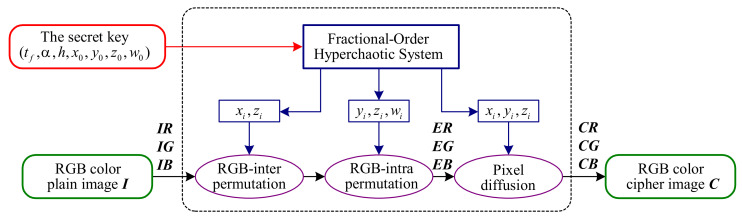
The block diagram of CIEA-FOHS.

As shown in Figure 2, CIEA-FOHS consists of three main parts: inter-permutation, intra-permutation and pixel diffusion. It is noted that, a two-dimensional image is transformed into an one-dimensional sequence in raster scan order. Specifically, a color plain image I of size H×W×3 is converted into three sequences of length H×W expressed as: IR, IG, and IB, which correspond to the three RGB channels of the image. The main contents are briefly introduced as follows:

- The Secret Key:

The secret keys of CIEA-FOHS include $\left(t_{f},\alpha ,h,x_{0},y_{0},z_{0},w_{0}\right)$, where $t_{f}$ is the fractional derivative defined by Caputo definition, α is the dimension, *h* is the step size for discretization, and $\left(x_{0},y_{0},z_{0},w_{0}\right)$ are the four initial values of the fractional-order hyperchaotic system defined in Equation (1), respectively. In CIEA-FOHS, these keys are used to generate some chaos-based pseudo-random sequences for encryption [42].

- Initialization:

In Equation (1), by selecting the secret key as the initial values and parameters and iterating *L* times, one gets four chaos-based pseudo-random sequences ${\left\{x_{i}\right\}}_{i=1}^{L}$, ${\left\{y_{i}\right\}}_{i=1}^{L}$, ${\left\{z_{i}\right\}}_{i=1}^{L}$ and ${\left\{w_{i}\right\}}_{i=1}^{L}$, where L=H×W represents the number of pixels in a single image channel.

- Stage 1. RGB-inter permutation:

The RGB-inter permutation refers to the process of pixel replacement between channels. This stage is implemented by two control vectors ${\left\{selE_{i}\right\}}_{i=1}^{L}$ and ${\left\{selLen_{i}\right\}}_{i=1}^{L}$, which are given as

(2) $$\left\{\begin{array}{c} selE_{i}=\left(\right|x_{i}\left|\times 10^{14}\right)\;\mathrm{mod}\;6\\ selLen_{i}=\left(\right|z_{i}\left|\times 10^{14}\right)\;\mathrm{mod}\;3 \end{array}\right.$$

where i=1∼L. More specifically, ${\left\{selE_{i}\right\}}_{i=1}^{L}$ is used to switch channels, as shown in Table 2, and ${\left\{selLen_{i}\right\}}_{i=1}^{L}$ is to control the position and length of the permutation pixel, given as

(3) $$\left\{\begin{array}{c} length=\left(sum\left(ER\left(pos:pos+length-1\right)\right)\;\mathrm{mod}\;64\right),ifselLen_{i}=0\\ length=\left(sum\left(EG\left(pos:pos+length-1\right)\right)\;\mathrm{mod}\;64\right),ifselLen_{i}=1\\ length=\left(sum\left(EB\left(pos:pos+length-1\right)\right)\;\mathrm{mod}\;64\right),ifselLen_{i}=2 \end{array}\right.$$

where pos is the starting position, length is the length of the permautation pixels, and sum is the cumulative function.

- Stage 2. RGB-intra permutation:

Sort ${\left\{y_{i}\right\}}_{i=1}^{L}$, ${\left\{z_{i}\right\}}_{i=1}^{L}$, and ${\left\{w_{i}\right\}}_{i=1}^{L}$ to get three index sequences ${\left\{IY_{i}\right\}}_{i=1}^{L}$, ${\left\{IZ_{i}\right\}}_{i=1}^{L}$, and ${\left\{IW_{i}\right\}}_{i=1}^{L}$ respectively, and their values range [1,L]. Use ${\left\{IY_{i}\right\}}_{i=1}^{L}$, ${\left\{IZ_{i}\right\}}_{i=1}^{L}$, and ${\left\{IW_{i}\right\}}_{i=1}^{L}$ to permute ER, EG and EB respectively, given as $ER_{i}=ER\left(IY_{i}\right)$, $EG_{i}=EG\left(IZ_{i}\right)$ and $EB_{i}=EB\left(IW_{i}\right)$.

- Stage 3. Pixel diffusion:

Perform pixel diffusion on ER, EG and EB, and then get three channels of the cipher image C. Exactly, the three channels CR, CG and CB are defined as

(4) $$\left\{\begin{array}{c} CR_{i}=SX_{i}⊕\left(\left(ER_{i}+SX_{i}\right)\;\mathrm{mod}\;256\right)⊕CR_{i-1}\\ CG_{i}=SY_{i}⊕\left(\left(EG_{i}+SY_{i}\right)\;\mathrm{mod}\;256\right)⊕CG_{i-1}\\ CB_{i}=SZ_{i}⊕\left(\left(EB_{i}+SZ_{i}\right)\;\mathrm{mod}\;256\right)⊕CB_{i-1} \end{array}\right.$$

where i=1∼L, ⊕ is bitwise XOR operation, mod represents modulo operation, and $CR_{0}=SX_{L}$, $CG_{0}=SY_{L}$, and $CB_{0}=SZ_{L}$. Here, three diffusion sequences SX, SY and SZ are generated by $SX_{i}=round\left(x_{i}\right)\times 10^{14}$, $SY_{i}=round\left(y_{i}\right)\times 10^{14}$ and $SZ_{i}=round\left(z_{i}\right)\times 10^{14}$ respectively, where round is a rounding operation on real numbers.

Decryption is the inverse of encryption and is not described in detail here.

## 3. Security Analysis of CIEA-FOHS

### 3.1. Preliminary Analysis of CIEA-FOHS

Referring to the basic assumptions of cryptanalysis, everything about the cryptosystem is public and only the secret key is unknown for attackers [13]. Chosen-plaintext attack is a common and powerful method of cryptanalysis. It assumes that attackers can arbitrarily choose the plaintext that is conducive to deciphering and obtain the corresponding ciphertext [12]. Under the scenario of chosen-plaintext attack, attackers can construct special plain images, such as all black and all white, and obtain the corresponding cipher images to analyze the target cipher.

**Figure 3:**
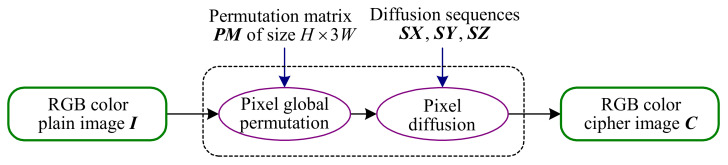
The block diagram of an equivalent simplified CIEA-FOHS.

From the perspective of cryptanalysis, two-stage permutations of CIEA-FOHS can be treated as a global pixel permutation because they only change the pixels’ position without their values. The difference is that the number of pixels performing the permutation is 3HW instead of HW. Then, the algorithm structure of CIEA-FOHS is actually a classic single-round permutation-diffusion. Moreover, the generation process of all chaos-based pseudo-random sequences is independent of the plain image, which means that these sequences can be regarded as an equivalent key. The reason is that, in the case of a given secret key, these sequences are fixed for encrypting different plain images with the same size. Then, CIEA-FOHS can be equivalently simplified as Figure 3, where PM is an equivalent permutation key and three diffusion sequences SX, SY and SZ serve as an equivalent diffusion key.

Based on the above, under the scenario of chosen-plaintext attack and the strategy of divide and conquer, one can get the equivalent keys and then recover the original plain images. Specifically, firstly choose some plain images with same pixel values to cancel the permutation and get the corresponding plain images to obtain the diffusion key; then achieve the permutation key by the method of Reference [12]; finally, recover the images by the equivalent keys.

### 3.2. Analysis on the Diffusion Part

In this section, based on chosen-plaintext attack, it is assumed that the plaintext image with the same pixel value is selected, and the corresponding ciphertext image is obtained.

- *Step 1.* Choose the all-zero plain image I${}^{\left(0\right)}$ and get the corresponding cipher image $C^{\left(0\right)}$ to determine $SX_{L},SY_{L},SZ_{L}$.

The reason for choosing the all-zero image is that the permutation is invalid at this time, and the diffusion can be eliminated to the greatest extent. Then, Equation (4) becomes

(5) $$\left\{\begin{array}{c} CR_{i}^{\left(0\right)}=CR_{i-1}^{\left(0\right)}\\ CG_{i}^{\left(0\right)}=CG_{i-1}^{\left(0\right)}\\ CB_{i}^{\left(0\right)}=CB_{i-1}^{\left(0\right)} \end{array}\right.$$

when i=1, one has $CR_{1}^{\left(0\right)}=CR_{0}$. Since $CR_{0}=SX_{L}$, thus $SX_{L}=CR_{i}^{\left(0\right)}$. Similarly, one further gets $SY_{L}=CG_{i}^{\left(0\right)}$ and $SZ_{L}=CB_{i}^{\left(0\right)}$.

- *Step 2.* Choose two special plain images and get the corresponding cipher images to determine $SX_{i},SY_{i},SZ_{i}$ for i=1∼L−1.

Referring to [43,44], the two chosen plaintexts are pure-color images with pixel values of 85 and 170, represented as I${}^{\left(85\right)}$ and I${}^{\left(170\right)}$, respectively. Because for the combined operation of module addition and bitwise XOR, choosing these two plain images can minimize the number of solutions for SX, SY, SZ. Under the plain image I${}^{\left(85\right)}$ and its corresponding cipher image C${}^{\left(85\right)}$, one gets

(6) $$\left\{\begin{array}{c} CR_{i}^{\left(85\right)}=SX_{i}⊕\left(\left(85+SX_{i}\right)\;\mathrm{mod}\;256\right)⊕CR_{i-1}^{\left(85\right)}\\ CG_{i}^{\left(85\right)}=SY_{i}⊕\left(\left(85+SY_{i}\right)\;\mathrm{mod}\;256\right)⊕CG_{i-1}^{\left(85\right)}\\ CB_{i}^{\left(85\right)}=SZ_{i}⊕\left(\left(85+SZ_{i}\right)\;\mathrm{mod}\;256\right)⊕CB_{i-1}^{\left(85\right)} \end{array}\right.$$

Similarly, given the plain image I${}^{\left(170\right)}$ and its corresponding cipher image $C^{\left(170\right)}$, one has

(7) $$\left\{\begin{array}{c} CR_{i}^{\left(170\right)}=SX_{i}⊕\left(\left(170+SX_{i}\right)\;\mathrm{mod}\;256\right)⊕CR_{i-1}^{\left(170\right)}\\ CG_{i}^{\left(170\right)}=SY_{i}⊕\left(\left(170+SY_{i}\right)\;\mathrm{mod}\;256\right)⊕CG_{i-1}^{\left(170\right)}\\ CB_{i}^{\left(170\right)}=SZ_{i}⊕\left(\left(170+SZ_{i}\right)\;\mathrm{mod}\;256\right)⊕CB_{i-1}^{\left(170\right)} \end{array}\right.$$

By performing bitwise on Equations (6) and (7), one further gets

(8) $$\left\{\begin{array}{c} \left(85\dot{+}SX_{i}\right)⊕\left(170\dot{+}SX_{i}\right)=CR_{i}^{\left(85\right)}⊕CR_{i-1}^{\left(85\right)}⊕CR_{i}^{\left(170\right)}⊕CR_{i-1}^{\left(170\right)}\\ \left(85\dot{+}SY_{i}\right)⊕\left(170\dot{+}SY_{i}\right)=CG_{i}^{\left(85\right)}⊕CG_{i-1}^{\left(85\right)}⊕CG_{i}^{\left(170\right)}⊕CG_{i-1}^{\left(170\right)}\\ \left(85\dot{+}SZ_{i}\right)⊕\left(170\dot{+}SZ_{i}\right)=CB_{i}^{\left(85\right)}⊕CB_{i-1}^{\left(85\right)}⊕CB_{i}^{\left(170\right)}⊕CB_{i-1}^{\left(170\right)} \end{array}\right.$$

where $\dot{+}$ is defined as $a\dot{+}b\overset{\Delta }{\;=}\;\mathrm{mod}\;\left(a+b,256\right)$. It is worth pointing out that the reason why 85 and 170 are chosen as the attack images is that their binary are 01010101 and 10101010 respectively. At this time, the number of possible solutions of $SX_{i},SY_{i},SZ_{i}$ is the smallest, which is two. More precisely, the difference between the two solutions is 128. Then, based on Equation (8), we propose Alogrithm 1 to determine $SX_{i},SY_{i},SZ_{i}$, where i=1∼L−1.

**Algorithm 1:** Determining $SX_{i},SY_{i},SZ_{i}$ for i=1∼L−1

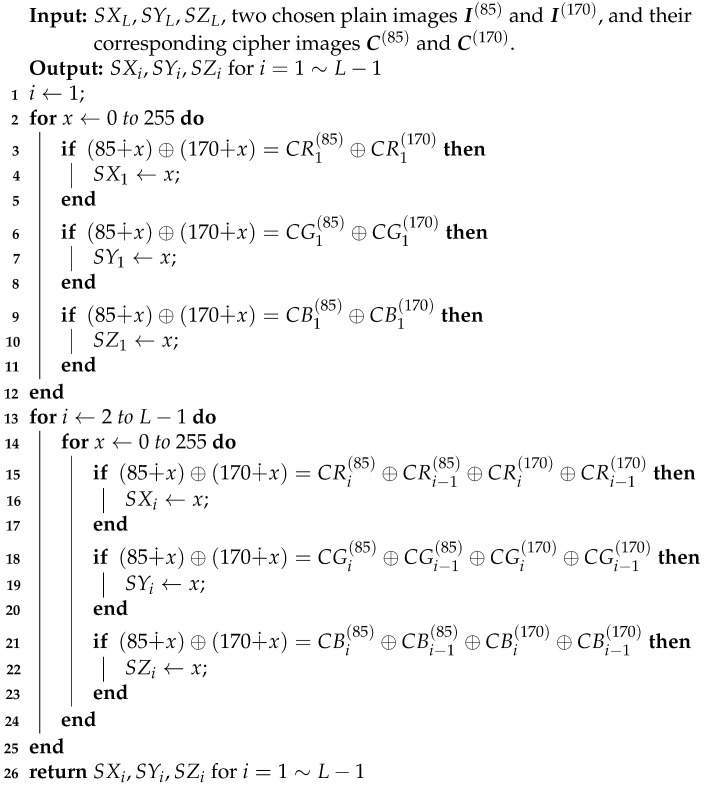

- *Step 3.* Eliminate the diffusion part by SX, SY, SZ.

Corresponding to Equation (4), the decryption process of diffusion is given as

(9) $$\left\{\begin{array}{c} ER_{i}=\left(SX_{i}⊕CR_{i}⊕CR_{i-1}-SX_{i}\right)\;\mathrm{mod}\;256\\ EG_{i}=\left(SY_{i}⊕CG_{i}⊕CG_{i-1}-SY_{i}\right)\;\mathrm{mod}\;256\\ EB_{i}=\left(SZ_{i}⊕CB_{i}⊕CB_{i-1}-SZ_{i}\right)\;\mathrm{mod}\;256 \end{array}\right.$$

Thus, ER, EG, EB can be restored from CR, CG, CB with SX, SY, SZ, respectively.

### 3.3. Analysis on the Permutation Part

Once the diffusion part is broken, CIEA-FOHS degenerates into a permutation-only cipher. Based on existing research, it cannot resist a chosen-plaintext attack. The basic idea of attacking permutation-only is to construct a special plain image with unequal element values, and get the corresponding permuted image. Taking 2×2×3 as an example, the process of solving PM is described below. First, a chosen plain image and the corresponding permuted image are given as

$$\mathrm{IR}=\left[\begin{array}{cc} 0 & 1\\ 2 & 3 \end{array}\right];\mathrm{IG}=\left[\begin{array}{cc} 4 & 5\\ 6 & 7 \end{array}\right];\mathrm{IB}=\left[\begin{array}{cc} 8 & 9\\ 10 & 11 \end{array}\right]$$

$$\mathrm{ER}=\left[\begin{array}{cc} 5 & 8\\ 3 & 11 \end{array}\right];\mathrm{EG}=\left[\begin{array}{cc} 1 & 10\\ 2 & 9 \end{array}\right];\mathrm{EB}=\left[\begin{array}{cc} 6 & 4\\ 0 & 7 \end{array}\right]$$

For ease of explanation, a matrix of size H×3W is obtained by connecting three channels of size H×W in a row connection manner. Then, the permutation process can be described by

$$\left[\begin{array}{cccccc} 0 & 1 & 4 & 5 & 8 & 9\\ 2 & 3 & 6 & 7 & 10 & 11 \end{array}\right]\overset{\mathrm{PM}}{\to }\left[\begin{array}{cccccc} 5 & 8 & 1 & 10 & 6 & 4\\ 3 & 11 & 2 & 9 & 0 & 7 \end{array}\right]$$

where PM is the permutation matrix of size H×3W. Finally, PM is determined as

(10) $$\mathrm{PM}=\left[\begin{array}{cccccc} \left(2,5\right) & \left(1,3\right) & \left(1,6\right) & \left(1,1\right) & \left(1,2\right) & \left(2,4\right)\\ \left(2,3\right) & \left(2,1\right) & \left(1,5\right) & \left(2,6\right) & \left(1,4\right) & \left(2,2\right) \end{array}\right]$$

Obviously, one can recover (IR,IG,IB) from (ER,EG,EB) with PM. However, the situation may be more complicated for large size images. For an 8-bit image, the pixel value range is [0,255]. Thus, when 3HW>256, PM cannot be determined by only one chosen plain image and its corresponding cipher image. Fortunately, this problem has been solved in our latest research [12,13]. The basic idea is to combine multiple chosen plain images in a weighted manner to form a matrix with different elements, and the number of chosen plain images required for attacking permutation is $\left\lceil \log_{256}\left(3HW\right)\right\rceil$, where $\left\lceil .\right\rceil$ is the rounding up operation.

Based on the above, the steps for attacking permutation are briefly summarized as follows:

- *Step 1.* Choose some special plain images and get their corresponding cipher images to determine the permutation matrix PM;
- *Step 2.* Use the permutation matrix PM to recover the original images from the permuted images.

### 3.4. The Proposed Chosen-Plaintext Attack Method

**Figure 4:**
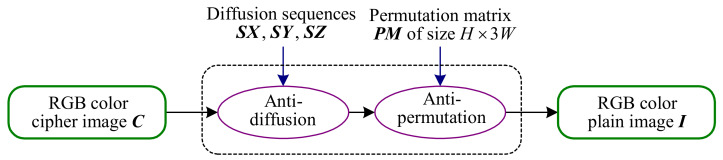
The overall flowchart of attacking CIEA-FOHS.

Following the above-mentioned discussion, CIEA-FOHS cannot resist the attack method proposed in this paper. The flowchart of the attack method is shown in Figure 4, and the specific steps based on chosen-plaintext attack are given as: firstly, get an equivalent diffusion key (SX,SY,SZ) by the method in Section 3.2; secondly, achieve the permutation matrix PM by the method in Section 3.3; finally, recover the original images with the equivalent keys.

Moreover, the complexity required for the attack method is discussed here. In terms of data complexity, for color images of size H×W×3, the number of chosen plain images required to decipher diffusion and permutation is 3 and $\left\lceil \log_{256}\left(3HW\right)\right\rceil$, respectively. Hence, the total data complexity required is $O(3+\left\lceil \log_{256}\left(3HW\right)\right\rceil )$.

## 4. Experimental Verifications and Discussions

To verify our security analysis, the algorithm steps of CIEA-FOHS strictly follow Ref. [42]. Although Due to the complexity of fractional-order chaos, some parameters may not be completely consistent, but this does not affect the effectiveness of security analysis. We conduct simulation verification on the proposed image cryptosystem based on a PC (personal computer) with MATLAB r2018b. The running PC is installed with Windows 10 64-bit OS (operating system), Intel(R) Core(TM) i5-8265U CPU @ 1.60 GHz and 8 GB memory. We select some typical images listed in Table 3 for experiments. Among them, the image “Lenna” of size 256×256×3 given in Ref. [42] is also included. In Equation (1), we set the experimental secret key parameters for *h* = 0.001, α = 104, $t_{f}$ = 100, $x_{0}$ = 1.002, $y_{0}$ = 0.949, $z_{0}$ = 0.997 and $w_{0}$ = 1.103.

**Figure 5:**
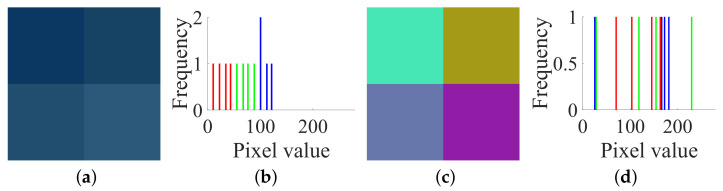
A pair of plain and cipher images of size 2×2×3: (**a**) plain image I; (**b**) histogram of I; (**c**) cipher image C; (**d**) histogram of C.

- *Case 1.* Breaking CIEA-FOHS with an image of size 2×2×3:

In order to better illustrate the attack process, we first adopt an extremely simple image with a size of 2 × 2 × 3. A pair of the given target plain and cipher images I and C is shown in Figure 5a,c respectively, and their histograms are shown in Figure 5b,d respectively. Accordingly, the numerical matrices of I and C are:

$$\mathrm{IR}=\left[\begin{array}{cc} 11 & 22\\ 33 & 44 \end{array}\right];\mathrm{IG}=\left[\begin{array}{cc} 55 & 66\\ 77 & 88 \end{array}\right];\mathrm{IB}=\left[\begin{array}{cc} 99 & 100\\ 111 & 122 \end{array}\right]$$

$$\mathrm{CR}=\left[\begin{array}{cc} 70 & 165\\ 103 & 145 \end{array}\right];\mathrm{CG}=\left[\begin{array}{cc} 231 & 154\\ 118 & 28 \end{array}\right];\mathrm{CB}=\left[\begin{array}{cc} 181 & 24\\ 171 & 165 \end{array}\right]$$

**Figure 6:**
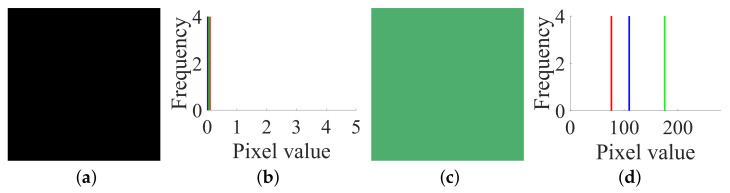
The all-zero chosen plain image $I^{\left(0\right)}$ and its corresponding cipher image $C^{\left(0\right)}$ of size 2×2×3: (**a**) $I^{\left(0\right)}$; (**b**) histogram of $I^{\left(0\right)}$; (**c**) $C^{\left(0\right)}$; (**d**) histogram of $C^{\left(0\right)}$.

Firstly, following Step 1 in Section 3.2, choose the all-zero plain image $I^{\left(0\right)}$ shown in Figure 6a and temporarily use the encryption machine of CIEA-FOHS, and then get the corresponding cipher image $C^{\left(0\right)}$, as shown in Figure 6c. The all-zero plain image $I^{\left(0\right)}$ and the corresponding cipher image $C^{\left(0\right)}$ and their histograms are shown in Figure 6b,d, respectively. Similarly, the numerical matrices of $I^{\left(0\right)}$ and $C^{\left(0\right)}$ are:

$${\mathrm{IR}}^{\left(0\right)}=\left[\begin{array}{cc} 0 & 0\\ 0 & 0 \end{array}\right];{\mathrm{IG}}^{\left(0\right)}=\left[\begin{array}{cc} 0 & 0\\ 0 & 0 \end{array}\right];{\mathrm{IB}}^{\left(0\right)}=\left[\begin{array}{cc} 0 & 0\\ 0 & 0 \end{array}\right]$$

$${\mathrm{CR}}^{\left(0\right)}=\left[\begin{array}{cc} 77 & 77\\ 77 & 77 \end{array}\right];{\mathrm{CG}}^{\left(0\right)}=\left[\begin{array}{cc} 174 & 174\\ 174 & 174 \end{array}\right];{\mathrm{CB}}^{\left(0\right)}=\left[\begin{array}{cc} 109 & 109\\ 109 & 109 \end{array}\right]$$

Then, one has $SX_{L}=77$, $SY_{L}=174$ and $SZ_{L}=109$ because $SX_{L}=CR_{0}$, $SY_{L}=CG_{0}$ and $SZ_{L}=CB_{0}$, where L=2×2=4.

**Figure 7:**
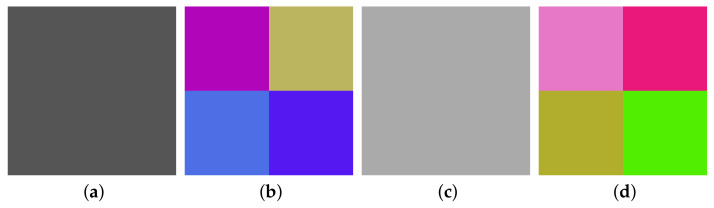
The two chosen plain images $I^{\left(85\right)}$, $I^{\left(170\right)}$ and their corresponding cipher images $C^{\left(85\right)}$, $C^{\left(170\right)}$ of size 2×2×3: (**a**) $I^{\left(85\right)}$; (**b**) $C^{\left(85\right)}$; (**c**) $I^{\left(170\right)}$; (**d**) $C^{\left(170\right)}$.

Secondly, based on Step 2 in Section 3.2, choose the two plain images $I^{\left(85\right)}$ and $I^{\left(170\right)}$, and get the corresponding cipher images, $C^{\left(85\right)}$ and $C^{\left(170\right)}$, which are shown in Figure 7a–d, respectively. The values of their RGB three channels are:

$${\mathrm{IR}}^{\left(85\right)}=\left[\begin{array}{cc} 85 & 85\\ 85 & 85 \end{array}\right];{\mathrm{IG}}^{\left(85\right)}=\left[\begin{array}{cc} 85 & 85\\ 85 & 85 \end{array}\right];{\mathrm{IB}}^{\left(85\right)}=\left[\begin{array}{cc} 85 & 85\\ 85 & 85 \end{array}\right]$$

$${\mathrm{CR}}^{\left(85\right)}=\left[\begin{array}{cc} 176 & 186\\ 77 & 85 \end{array}\right];{\mathrm{CG}}^{\left(85\right)}=\left[\begin{array}{cc} 5 & 181\\ 110 & 24 \end{array}\right];{\mathrm{CB}}^{\left(85\right)}=\left[\begin{array}{cc} 184 & 94\\ 229 & 241 \end{array}\right]$$

$${\mathrm{IR}}^{\left(170\right)}=\left[\begin{array}{cc} 170 & 170\\ 170 & 170 \end{array}\right];{\mathrm{IG}}^{\left(170\right)}=\left[\begin{array}{cc} 170 & 170\\ 170 & 170 \end{array}\right];{\mathrm{IB}}^{\left(170\right)}=\left[\begin{array}{cc} 170 & 170\\ 170 & 170 \end{array}\right]$$

$${\mathrm{CR}}^{\left(170\right)}=\left[\begin{array}{cc} 231 & 235\\ 177 & 81 \end{array}\right];{\mathrm{CG}}^{\left(170\right)}=\left[\begin{array}{cc} 120 & 24\\ 174 & 238 \end{array}\right];{\mathrm{CB}}^{\left(170\right)}=\left[\begin{array}{cc} 199 & 123\\ 45 & 1 \end{array}\right]$$

Then, combining Algorithm 1, we determine SX SY SZ as

$$\mathrm{SX}=\left[\begin{array}{cccc} 84 & 86 & 89 & 77 \end{array}\right];\mathrm{SY}=\left[\begin{array}{cccc} 63 & 31 & 71 & 46 \end{array}\right];\mathrm{SZ}=\left[\begin{array}{cccc} 64 & 36 & 119 & 109 \end{array}\right]$$

or

$$\mathrm{SX}=\left[\begin{array}{cccc} 212 & 214 & 217 & 205 \end{array}\right];\mathrm{SY}=\left[\begin{array}{cccc} 191 & 159 & 199 & 174 \end{array}\right];\mathrm{SZ}=\left[\begin{array}{cccc} 192 & 164 & 247 & 237 \end{array}\right]$$

**Figure 8:**
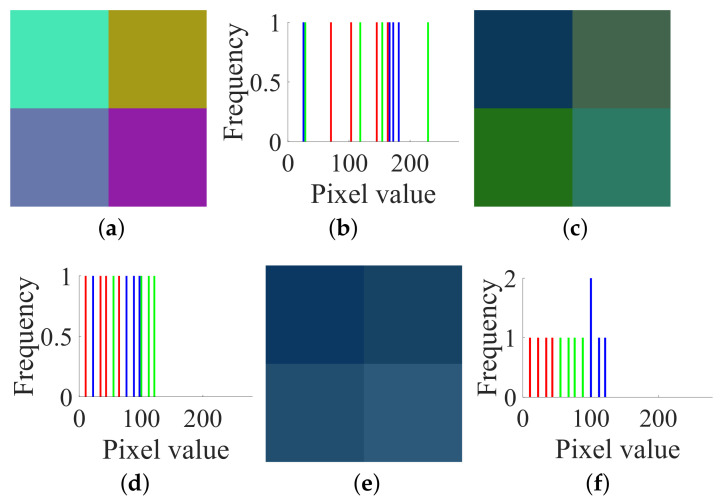
A target cipher image, the permuted image, the original plain image and their histograms of size 2×2×3: (**a**) a target cipher image; (**b**) histogram of (**a**); (**c**) its permuted image; (**d**) histogram of (**c**); (**e**) its plain image; (**f**) histogram of (**e**).

Thirdly, by Step 3 in Section 3.2, the corresponding permuted image shown in Figure 8c can be restored from the targeted cipher image Figure 8a with SX SY SZ. Fourthly, following Step 1 in Section 3.3, construct some special attack images to obtain the permutation matrix PM. For images of size 2×2×3, the process of solving PM is exactly the same as Section 3.3. Then, we determine the PM as Equation (10). Fifth, by Step 2 in Section 3.3, recover (IR,IG,IB) from (ER,EG,EB) with PM. Thus, the original plain image shown in Figure 8e can be recovered.

**Figure 9:**
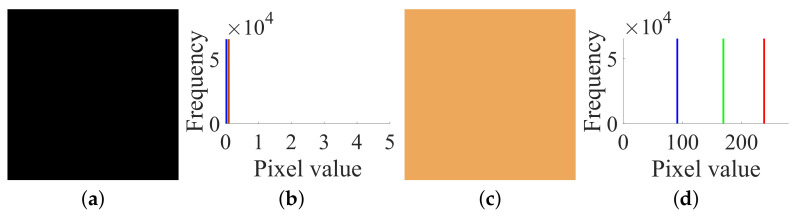
The all-zero chosen plain image $I^{\left(0\right)}$ and its corresponding cipher image $C^{\left(0\right)}$ of size 256×256×3: (**a**) $I^{\left(0\right)}$; (**b**) histogram of $I^{\left(0\right)}$; (**c**) $C^{\left(0\right)}$; (**d**) histogram of $C^{\left(0\right)}$.

- *Case 2.* Breaking CIEA-FOHS with “Lenna” of size 256×256×3:

Firstly, following Step 1 in Section 3.2, choose the all-zero plain image $I^{\left(0\right)}$ shown in Figure 9a and temporarily use the encryption machine of CIEA-FOHS, and then get the corresponding cipher image $C^{\left(0\right)}$, as shown in Figure 9b, and the corresponding three channel images and their histograms of $C^{\left(0\right)}$ are shown in Figure 9c,d, respectively. Exactly, one has $SX_{L}=238$, $SY_{L}=168$ and $SZ_{L}=91$ owing to $SX_{L}=CR_{0}$, $SY_{L}=CG_{0}$ and $SZ_{L}=CB_{0}$.

**Figure 10:**
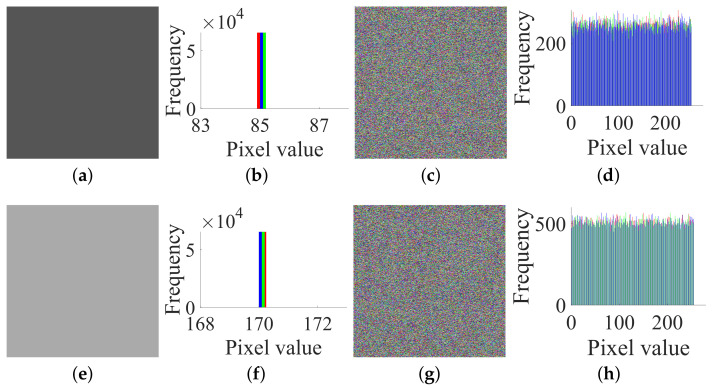
The two chosen plain images $I^{\left(85\right)}$, $I^{\left(170\right)}$ and their corresponding cipher images $C^{\left(85\right)}$, $C^{\left(170\right)}$ of size 256×256×3: (**a**) $I^{\left(85\right)}$; (**b**) histogram of $I^{\left(85\right)}$; (**c**) $C^{\left(85\right)}$; (**d**) histogram of $C^{\left(85\right)}$; (**e**) $I^{\left(170\right)}$; (**f**) histogram of $I^{\left(170\right)}$; (**g**) $C^{\left(170\right)}$; (**h**) histogram of $C^{\left(170\right)}$.

Secondly, based on Step 2 in Section 3.2, choose the two plain images, $I^{\left(85\right)}$ and $I^{\left(170\right)}$, and get the corresponding cipher images, $C^{\left(85\right)}$ and $C^{\left(170\right)}$, which are shown in Figure 10a–d, respectively.

Furthermore, one determines $SX_{i},SY_{i},SZ_{i}$ for i=1∼L−1 by Algorithm 1.

**Figure 11:**
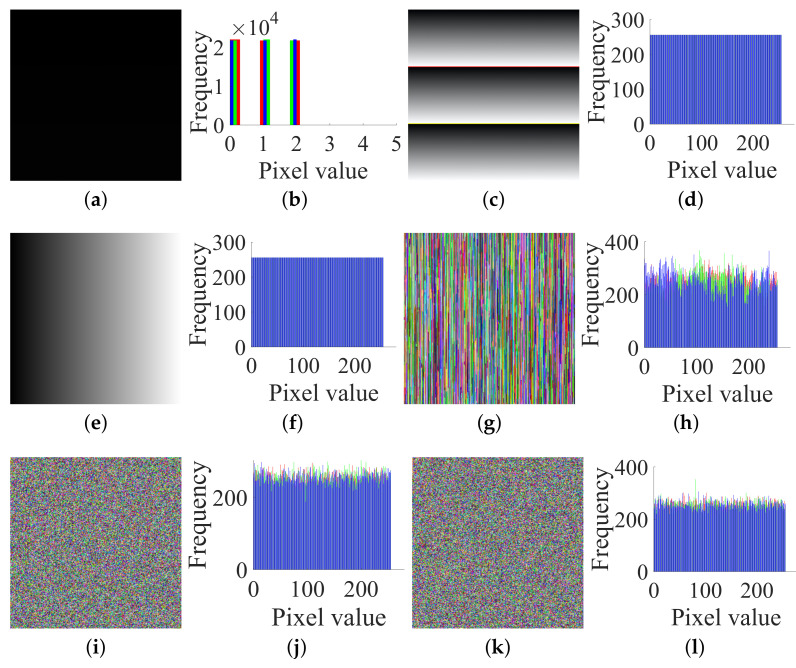

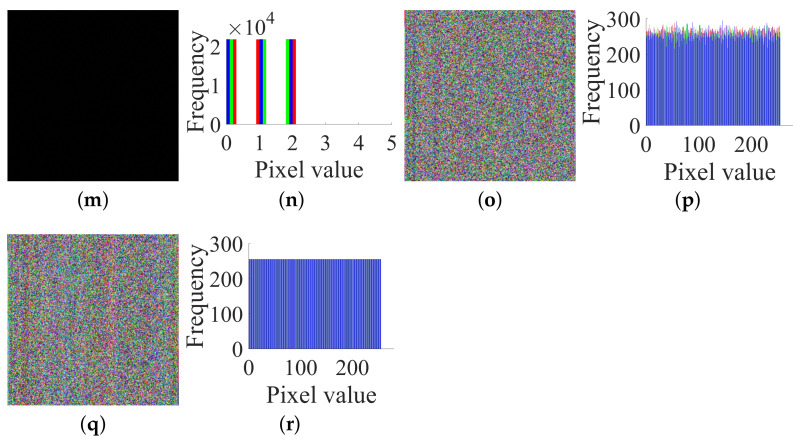
Three chosen plain images, the corresponding cipher and permuted images for attacking permutation: (**a**) $1^{\#}$ plain image; (**b**) The histogram of (**a**); (**c**) $2^{\#}$ plain image; (**d**) The histogram of (**c**); (**e**) $3^{\#}$ plain image; (**f**) The histogram of (**e**); (**g**) $1^{\#}$ cipher image; (**h**) The histogram of (**g**); (**i**) $2^{\#}$ cipher image; (**j**) The histogram of (**i**); (**k**) $3^{\#}$ cipher image; (**l**) The histogram of (**k**); (**m**) $1^{\#}$ permuted image; (**n**) The histogram of (**m**); (**o**) $2^{\#}$ permuted image; (**p**) The histogram of (**o**); (**q**) $3^{\#}$ permuted image; (**r**) The histogram of (**q**).

Thirdly, by the method in Section 3.3, choose the three plain images (shown in Figure 11a–f) and get the corresponding cipher images (shown in Figure 11g–l), and then use Algorithm 1 again to obtain their corresponding permuted images (shown in Figure 11m–r). Then, we can get PM.

**Figure 12:**
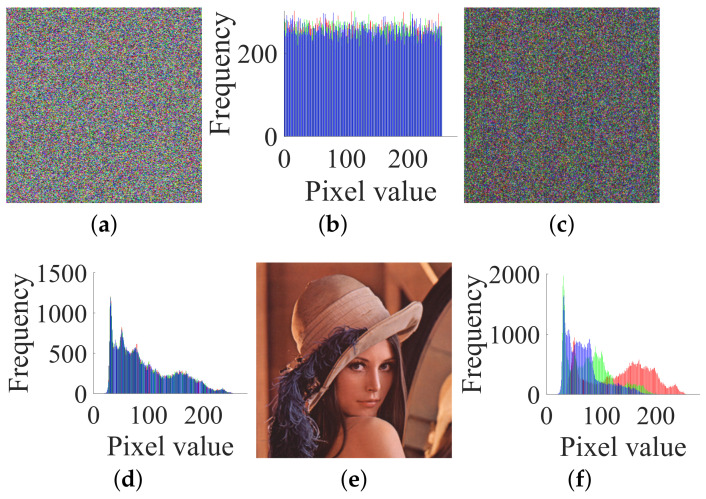
The cipher image, the permuted image, the original plain image of “Lenna” and their histograms of size 256×256×3: (**a**) the cipher image; (**b**) histogram of (**a**); (**c**) its permuted image; (**d**) histogram of (**c**); (**e**) its plain image; (**f**) histogram of (**e**).

Finally, we recover the original image from the cipher image of “Lenna” shown in Figure 12a. First, the permuted image shown in Figure 12c is obtained from the cipher image with (SX, SY, SZ). Then, the plain image is restored by PM, which is shown in Figure 12e.

**Figure 13:**
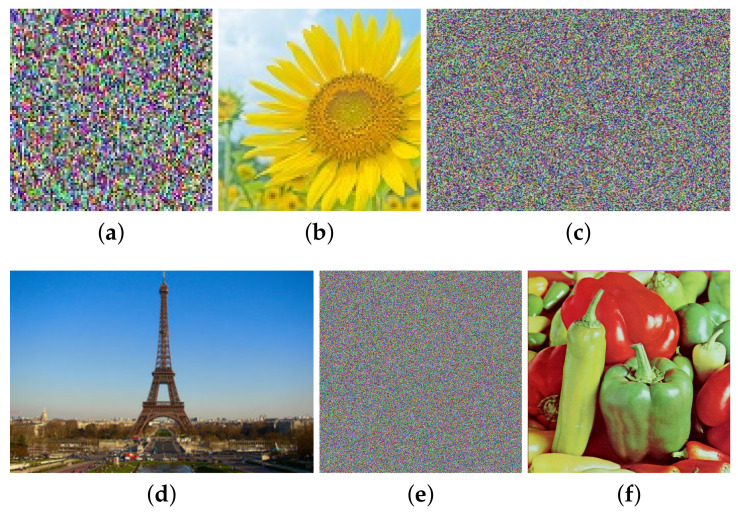
Attacking results with three images of size 100×100×3, 300×200×3 and 512×512×3 respectively: (**a**) cipher image of size 100×100×3; (**b**) plain image of (**a**); (**c**) cipher image of size 300×200×3; (**d**) plain image of (**c**); (**e**) cipher image of size 512×512×3; (**f**) plain image of (**e**).

Without loss of generality, we do the experiments based on other images with different sizes. The experimental results are shown in Table 3 and Figure 13. They both verify the effectiveness of our attack method. Besides, it can be seen from Table 3 that the proposed attack is efficient. Taking the image “Lenna” of size 256×256×3 as an example, when the encryption time is 0.6391 s, the time needed for the corresponding attack is just 129.4039 s. Even if the image size increases, the time required for the attack is still within an acceptable range. Thus, it verifies that our method is computationally feasible.

Moreover, we verified the data complexity required for the attack. As discussed in Section 3.4, the total data complexity required for breaking CIEA-FOHS is $O(3+\left\lceil \log_{256}\left(3HW\right)\right\rceil )$. In our experiment with chosen-plaintext attack, the number of attack images required for sizes 2×2×3 and 100×100×3 are 4 and 5, respectively. And for sizes 300×200×3, 256×256×3 and 512×512×3, the number of attack images required are all 6. Therefore, the experimental verification is consistent with the theoretical calculation.

## 5. Suggestions for Improvement

On the basis of the above, CIEA-FOHS is insecure against a chosen-plaintext attack method because of its inherent security defects. To enhance the security, some suggestions for improvement are listed below:

- Suggestion 1. Ensuring the substantial security contribution of the fractional-order chaos to the corresponding cipher. The attractor phase diagram of the fractional-order hyperchaotic system is shown in Figure 1, which shows the extremely complex dynamics. Undoubtedly, fractional-order chaos is one of the preferred sources of entropy for encryption. However, due to the negligence of algorithm design, CIEA-FOHS has serious security defects and is attacked.
- Suggestion 2. Security analysis should be implemented from the perspective of cryptography, not limited to numerical statistical verification. As Ref. [45] points out, many encryption algorithms have excellent statistical analysis results, but they are still insecure. In fact, good statistical analysis results are only a necessary and not a sufficient condition for security. Some security flaws are difficult to reflect with numerical statistical results, but they can be clearly revealed by theoretical security analysis. For example, the existence of an equivalent key makes CIEA-FOHS vulnerable to cryptographic attacks. Given the implementation of detailed cryptographic security analysis, these flaws can be avoided, thereby improving security.

## 6. Conclusions

In this paper, a detailed security analysis of a color image encryption algorithm named CIEA-FOHS using a fractional-order chaos was performed. From the perspective of cryptanalysis, this paper found that CIEA-FOHS can be broken by a chosen-plaintext attack method, owing to its some inherent security defects. Theoretical analysis and experimental simulations show that the attack method is both effective and efficient for attacking CIEA-FOHS. Although the fractional-order chaotic system has complex dynamics, the algorithm defects may cause insecurity. The reported results would help the designers of chaotic cryptography pay more attention to the gap between complex chaotic system and secure cryptosystem.

## Figures and Tables

**Table 1:** Some chaos-based ciphers broken by various attack methods.

Ciphers	Broken by	Attack Methods
Fridrich et al. [34] in 1998	Xie et al. [16] in 2017	Chosen-ciphertext attack
Zhao et al. [35] in 2015	Norouzi et al. [36] in 2017	Chosen-plaintext attack
Ye [37] in 2010	Li et al. [18] in 2017	Cipher-only attack
Zhou [38] in 2015	Chen et al. [17] in 2016	Differential cryptanalysis
Song et al. [15] in 2015	Wen et al. [13] in 2019	Chosen-plaintext/cipertext attacks
Shafique et al. [14] in 2018	Wen et al. [12] in 2019	Chosen-plaintext attack

**Table 2:** The stutas of RGB-inter permutation under six rules.

Rule selE(i)	0	1	2	3	4	5
	R→R	R→R	R→G	R→B	R→G	R→B
Permutation status	G→G	G→B	G→R	G→R	G→B	G→G
	B→B	B→G	B→B	B→G	B→R	B→R

**Table 3:** The time required for breaking CIEA-FOHS by our proposed attack method (unit: second).

Images	Sizes	Encrytion Time	Attacking Diffusion			Attacking Permutation		Totol Attacking Time
			*Step* 1	*Step* 2	*Step* 3	*Step* 1	*Step* 2	
Figure 5a	2 × 2 × 3	0.0280	0.1559	0.1811	1.0297	0.0244	2.7151	4.1502
Figure 13b	100 × 100 × 3	0.1539	0.0920	19.6092	1.1407	0.2764	2.7102	24.0427
Figure 13d	300 × 200 × 3	0.3280	0.5092	101.7737	0.7872	0.9055	2.4353	106.8545
Figure 12e	256 × 256 × 3	0.6391	0.6913	120.4768	1.6147	1.9642	3.7725	129.4039
Figure 13f	512 × 512 × 3	3.5386	2.8134	988.3704	1.9930	4.2884	5.0459	1004.4617

## Data Availability

Not applicable.
